# Diagnostic Performance of the Radiographic Assessment of Lung Edema Score for Acute Respiratory Distress Syndrome—Post Hoc Analysis of the “Validating Acute Lung Injury Biomarkers for Diagnosis” Study

**DOI:** 10.1097/CCE.0000000000001495

**Published:** 2026-10-21

**Authors:** Claudio Zimatore, Matthieu Jabaudon, Luigi Pisani, Tatsuki Koyama, Marcus J. Schultz, Lorraine B. Ware

**Affiliations:** 1 Department of Intensive Care, Amsterdam University Medical Centers, Amsterdam, The Netherlands.; 2 Division of Intensive Care, Department of Precision and Regenerative Medicine and Ionian Area (DiMePRe-j), University of Bari “Aldo Moro,” Bari, Italy.; 3 Department of Perioperative Medicine, Centre Hospitalier Universitaire (CHU) Clermont-Ferrand, Clermont-Ferrand, France.; 4 iGReD, CNRS, INSERM, Université Clermont Auvergne, Clermont-Ferrand, France.; 5 Mahidol–Oxford Tropical Medicine Research Unit (MORU), Mahidol University, Bangkok, Thailand.; 6 Department of Biostatistics, Vanderbilt University Medical Center, Nashville, TN.; 7 Nuffield Department of Medicine, University of Oxford, Oxford, United Kingdom.; 8 Department of Anesthesia, General Intensive Care and Pain Management, Division of Cardiothoracic and Vascular Anesthesia & Critical Care Medicine, Medical University of Vienna, Vienna, Austria.; 9 Department of Medicine, Division of Allergy, Pulmonary and Critical Care Medicine, Vanderbilt University Medical Center, Nashville, TN.; 10 Department of Pathology, Microbiology and Immunology, Vanderbilt University Medical Center, Nashville, TN.

**Keywords:** acute respiratory distress syndrome, chest radiograph, chest x-ray, critical care, diagnostic accuracy, intensive care, intraclass correlation coefficient, invasive ventilation, lung imaging, Radiographic Assessment of Lung Edema score

## Abstract

**OBJECTIVES:**

To determine the diagnostic accuracy of the Radiographic Assessment of Lung Edema (RALE) score for acute respiratory distress syndrome (ARDS) and its interobserver reliability.

**DESIGN:**

Post hoc analysis of a prospective observational cohort study.

**SETTING:**

ICUs at Vanderbilt University Medical Center participating in the Validating Acute Lung Injury Biomarkers for Diagnosis study.

**PATIENTS:**

Adult patients receiving invasive ventilation for at least two consecutive days, with chest radiographs available on the first 2 ICU days.

**INTERVENTIONS:**

None.

**MEASUREMENTS AND MAIN RESULTS:**

Chest radiographs from the first 2 ICU days were scored independently by at least two experienced raters; scores were averaged per radiograph, and the highest averaged score from ICU day 1 or 2 was selected for analysis. The primary outcome was ARDS according to the Berlin definition during the first 2 ICU days. Of 236 patients, 92 (39%) had ARDS. The median RALE score was higher in patients with ARDS than in those without (29.5 [22.2–34.5] vs. 12.2 [7.5–17.8]; *p* < 0.01). The area under the receiver operating characteristic curve was 0.93 (95% CI, 0.90–0.96). At the optimal cutoff of 20, sensitivity was 85%, specificity 83%, positive predictive value 76%, and negative predictive value 90%. Interobserver reliability was high, with pairwise intraclass correlation coefficients ranging from 0.81 to 0.87.

**CONCLUSIONS:**

In patients invasively ventilated for at least 2 days, the RALE score demonstrated excellent diagnostic accuracy for ARDS at a cutoff of 20, together with high interobserver reliability, supporting its potential role as a standardized radiographic tool in the diagnosis of ARDS.

**KEY POINTS**

**Question:** Can the Radiographic Assessment of Lung Edema (RALE) score identify acute respiratory distress syndrome (ARDS) in invasively ventilated patients?

**Findings:** Among 236 patients, of whom 92 had ARDS, the RALE score discriminated ARDS with an area under the receiver operating characteristic curve of 0.93; a cutoff of 20 yielded 85% sensitivity and 83% specificity, with high interobserver reliability (pairwise intraclass correlation coefficients, 0.81–0.87).

**Meaning:** The RALE score offers a quantitative, reproducible measure of the radiographic abnormalities used to define ARDS; prospective, multimodal, and potentially automated validation is the logical next step toward its use within future diagnostic criteria.

Bilateral alveolar opacities on chest radiographs (CXRs) are a defining feature of acute respiratory distress syndrome (ARDS) (1). Their identification, however, is poorly reproducible even among trained clinicians (2, 3), resting on a subjective judgment influenced by radiograph quality, training, and experience, with consequences for both diagnosis and treatment. CXRs are widely accessible and routinely obtained in critically ill patients (4, 5), and, despite well-recognized limitations in depicting lung pathology (6), remain central to all past and current ARDS definitions (7). Standardized approaches to their interpretation are therefore needed.

The “Radiographic Assessment of Lung Edema” (RALE) score is a semi-quantitative system developed to quantify the extent and density of pulmonary edema on CXRs (8). It was not designed as a diagnostic tool for ARDS, and studies of its diagnostic accuracy remain scarce: one single-center study reported excellent accuracy (9), whereas reported interobserver agreement has ranged from moderate to excellent (8, 9). This variability highlights the challenges in implementing scoring systems across diverse clinical settings and suggests that additional validation studies may be needed to fully assess the reliability of RALE scoring.

This post hoc analysis evaluated the diagnostic accuracy of the RALE score for ARDS in the “Validating Acute Lung Injury Biomarkers for Diagnosis” (VALID) study, a prospective cohort of critically ill patients receiving invasive ventilation for at least 2 days, a substantial proportion of whom had ARDS (10). We hypothesized that the RALE score would show high diagnostic accuracy for ARDS, and we additionally assessed its interobserver reliability.

## MATERIALS AND METHODS

### Study Design and Settings

This was a post hoc analysis of VALID, a single-center, multi-unit, prospective observational study that was primarily designed to investigate panels of novel and established plasma, serum, and urine protein biomarkers to aid in diagnosing ARDS in patients at risk of this complication of critical illness (10). VALID was performed at the medical, cardiac, surgical, and trauma ICUs at Vanderbilt University Medical Center (VUMC), Nashville, TN. The methods for VALID have been previously published (10). The protocol of VALID, entitled “Validating Acute Lung Injury Biomarkers for Diagnosis”, was approved on November 11, 2005, by the Institutional Review Board (IRB) of VUMC (protocol number 051065; most recent continuing review approved on November 18, 2025). All procedures were performed in accordance with the ethical standards of the responsible institutional committee on human experimentation and with the Declaration of Helsinki 1975, as revised in 2013. Written informed consent was obtained from patients or their legally authorized representatives before enrollment. If patients were unable to consent and no surrogates had been identified, the IRB granted a waiver of informed consent for this minimal risk study. The present post hoc analysis used de-identified data from the VALID cohort, and all CXRs were scored at VUMC; no additional IRB approval was required. This analysis was reported in accordance with the Standards for Reporting of Diagnostic Accuracy Studies 2015 (**Table S1**, https://links.lww.com/CCX/B693).

### Patients

Consecutive adult patients of 18 years or older admitted to one of the four ICUs at VUMC from January 2006 onward were screened for inclusion in the VALID study. To be eligible, patients were required to have an ICU stay of at least 2 days. Exclusion criteria included cardiac arrest before enrollment, anticipated transfer or transfer orders within 4 hours, death or discharge within 48 hours of ICU admission, admission for uncomplicated overdose, an ICU stay exceeding 3 days before enrollment, and pre-existing chronic lung disease requiring oxygen therapy or pulmonary fibrosis.

For the current post hoc analysis, patients were eligible if they met two additional criteria: they received invasive ventilation for at least 2 days, starting from their first day in the ICU, and had a risk factor for ARDS. Both criteria were prespecified to define a homogeneous population of invasively ventilated patients at risk for ARDS. Patients were additionally excluded if they were admitted after traumatic injury, developed ARDS after the first 2 days of mechanical ventilation, or had clinical or radiographic evidence of cardiogenic pulmonary edema. Traumatic injury was excluded a priori because pulmonary contusion produces alveolar opacification indistinguishable from lung edema on a supine anteroposterior radiograph and would therefore raise the RALE score independently of the construct it is intended to quantify. No radiograph was excluded, and no patient was selected, based on the distribution or laterality of opacities; the RALE score was computed on every usable radiograph irrespective of whether opacities were unilateral or bilateral, and the classification of ARDS was made independently using the full Berlin definition.

### Data Collected and Clinical Outcomes

Collection of data involved patient demographics and baseline characteristics, including age, sex, height, weight, and body mass index; disease severity scores including the Acute Physiology and Chronic Health Evaluation (APACHE) II score and the Simplified Acute Physiology Score II. Ventilator-free days at day 28 (VFD-28) were calculated as the number of days within the first 28 days after ICU admission during which the patient remained alive and free from invasive ventilation. Patients who died before day 28 were assigned zero VFDs. Length of stay was defined as the number of days from ICU admission to ICU discharge.

### RALE Score

For each patient, all CXRs obtained during the first 2 days of ventilation were independently scored by at least two investigators with experience using the RALE score (rater I, rater II, and rater III). Each scorer rated the quality of the CXRs as acceptable, borderline (due to factors like poor image quality or interfering conditions such as pleural effusion or subcutaneous emphysema), or unusable (due to very poor image quality). CXRs were excluded from the analysis if any scorer classified them as unusable. All the remaining radiographs were scored by investigators who were blinded to all clinical data, including the presence or absence of ARDS, the PaO_2_/FiO_2_ ratio, the predisposing risk factor, and clinical outcomes, and who were unaware of group allocation; radiographs from patients with and without ARDS were presented intermixed. Raters were necessarily aware of the general aim of the project, the quantification of radiographic lung edema in an ICU cohort, but not of the classification of any individual patient. Conversely, ARDS was adjudicated within VALID by independent physicians applying the Berlin definition without reference to the RALE score. For each patient, scores from individual raters were first averaged for each radiograph; the CXR with the highest average score from either ICU day 1 or 2 was then selected.

To calculate the RALE score, the lung fields on the CXR were divided into four quadrants by a vertical line over the spine and a horizontal line at the level of the first branch of the left main bronchus, as outlined in the original RALE score publication (8). Each quadrant was scored based on the extent of alveolar opacities (the consolidation score, from 0 to 4) and the density of alveolar opacities (the density score, from 1 to 3). If the consolidation score was 0, the density score was also 0. The RALE score was calculated as the sum of the products of the consolidation and density scores for each quadrant, giving a total score from 0 to 48. Any CXR with substantial discrepancies in RALE score between two readers (a total score difference of 16 or more; or quadrant scores differing by three or more) was resolved through reconciliation between readers, as done in previous studies (11). Discrepancies were addressed by having all reviewers reassess the CXRs. The proportion of radiographs meeting these predefined discrepancy thresholds is reported in the Results. Reconciled values were used for the diagnostic analysis; the interobserver reliability analysis was based exclusively on the raters’ independent scores, obtained before any reconciliation.

### Endpoint

The endpoint of this post hoc analysis was ARDS, according to the Berlin definition, on the first 2 days of mechanical ventilation.

### Statistical Analysis

The prevalence of ARDS in VALID was estimated to be 40%, based on data from two previous studies (9, 12). With a two-sided type I error rate of 5%, this post hoc analysis had 80% power to detect an association between a dichotomized RALE score and the likelihood of ARDS. If the true probabilities of ARDS in the low and high RALE groups are 20% and 40%, respectively, a total sample size of 228 would provide this power. To account for potentially unusable CXRs, a total of 239 patients were required for the current analysis. Eligible patients were selected sequentially from the VALID database until the target sample size was reached.

Patient demographics, baseline characteristics, and outcomes are presented as proportions for categorical variables, and as medians with quartiles for continuous variables, reflecting the distribution of the data.

Continuous variables were compared using the Wilcoxon rank-sum test, and categorical variables were compared with Fisher exact test.

Highest average RALE scores are presented in box-and-whisker plots, showing medians, quartiles, ranges, and the scores for each patient with or without ARDS. In addition, RALE scores in the left and right lung are presented in plots for comparing the side with the higher value to the side with the lower score. The regional distribution of the RALE score between hemithoraces was examined descriptively; no statistical comparisons of regional scores were performed.

To determine the diagnostic accuracy of the RALE score for ARDS, receiver operating characteristic (ROC) curves were constructed, and the area under the receiver operating characteristic curve (AUROC) was calculated. Diagnostic accuracy was classified as “excellent” if the AUROC was between 0.9 and 1, “very good” between 0.8 and 0.9, “good” between 0.7 and 0.8, “sufficient” between 0.6 and 0.7, and “poor” between 0.5 and 0.6 (13). When determining the best cutoff value, we only considered integer values, as the RALE score itself is integer-based. Sensitivity, specificity, and positive and negative predictive values were then calculated using this cutoff (**Table S2**, https://links.lww.com/CCX/B693).

A sensitivity analysis was performed to test the robustness of the findings. This analysis included only patients with CXRs rated as “acceptable” by all scorers.

Finally, interobserver reliability was assessed using intraclass correlation coefficients (ICCs). Most CXRs were independently scored by only two raters, and a subset by all three raters. This design allowed for both pairwise comparisons between each pair of raters and overall agreement analysis among all three raters for the same image. ICCs were computed for each rater pair using a two-way mixed-effects model, assessing absolute agreement for both single and average measurements, as recommended for reliability studies. In addition, an overall ICC was calculated for the CXRs scored by all three raters (14). All ICCs were computed on the raters’ independent scores, obtained before any reconciliation of discrepant radiographs. ICC was classified as excellent (0.75–1.00), good (0.50–0.74), moderate (0.25–0.49), or poor (0.00–0.24).

All analyses were performed using R version 4.4 (R Core Team, R Foundation for Statistical Computing, Vienna, Austria). Statistical significance was considered when the *p* value was less than 0.05.

## RESULTS

### Patients

Of 239 sequentially selected patients, three patients were excluded due to unusable CXRs (**Fig. 1**). Ninety-two patients (39.0%) had ARDS, which was most often due to pneumonia, aspiration, or sepsis (**Table 1**). ARDS patients had a higher median APACHE II score and lower PaO_2_/FiO_2_. The number of CXRs per patient was high, but not different between patients with and without ARDS. ARDS patients stayed longer in the ICU, had fewer VFD-28, and had a higher mortality.

**TABLE 1.** Patient Demographics, Baseline Characteristics, and Outcomes

Characteristic	Patients With ARDS (*n* = 92)	Patients Without ARDS (*n* = 144)	*p*
Age, yr	56.0 (38.8–66.0)	58.0 (49.0–68.0)	0.12
Sex, female	35 (38)	61 (42)	0.59
Acute Physiology and Chronic Health Evaluation II Score	31.0 (27.0–38.0)	28.0 (24.0–34.2)	< 0.01
Simplified Acute Physiology Score II	62.5 (52.0–74.2)	59.0 (45.0–71.0)	0.07
Admission source			< 0.01
Emergency department	30 (33)	50 (35)	
Outside of hospital	22 (24)	51 (35)	
Floor	31 (34)	18 (13)	
Operating room	4 (4)	9 (6)	
Missing/other	5 (5)	16 (11)	
Risk factors for ARDS			< 0.01
Pneumonia	33 (36)	24 (17)	
Sepsis	22 (24)	67 (47)	
Aspiration	33 (36)	18 (13)	
Multiple transfusions	0 (0)	13 (9)	
Other	4 (4)	22 (15)	
Admission PaO_2_/FiO_2_, mm Hg	107 (67–154)	198 (101–264)	< 0.01
PaO_2_/FiO_2_ classification			< 0.01
> 300 mm Hg	0 (0)	26 (19)	
201–300 mm Hg	11 (12)	39 (29)	
101–200 mm Hg	42 (46)	35 (26)	
≤ 100 mm Hg	39 (42)	34 (25)	
Number of chest radiographs, mean ± sd	2.8 ± 1.5	2.4 ± 1.2	0.11
1	18 (20)	38 (26)	
2	24 (26)	44 (31)	
3	25 (27)	36 (25)	
4	17 (18)	13 (9)	
5 or more	8 (9)	13 (9)	
Chest radiographs, borderline			0.64
0	79 (86)	117 (81)	
1	8 (9)	20 (14)	
2	4 (4)	6 (4)	
3	1 (1)	1 (1)	
Clinical outcomes			
ICU length of stay, d	7.0 (4.0–11.0)	6.0 (3.0–8.0)	0.02
Ventilator-free days and alive at day 28	11.5 (1.0–24.0)	24.0 (10.8–26.0)	< 0.01
In-hospital mortality	38 (41)	32 (22)	< 0.01

ARDS = acute respiratory distress syndrome.

Data are presented as median (interquartile range) or *n* (%), unless stated otherwise. Risk factors are mutually exclusive and denote the single dominant predisposing condition; multiple transfusions denote the receipt of 10 units of RBCs within 24 hr.

**Figure 1.**
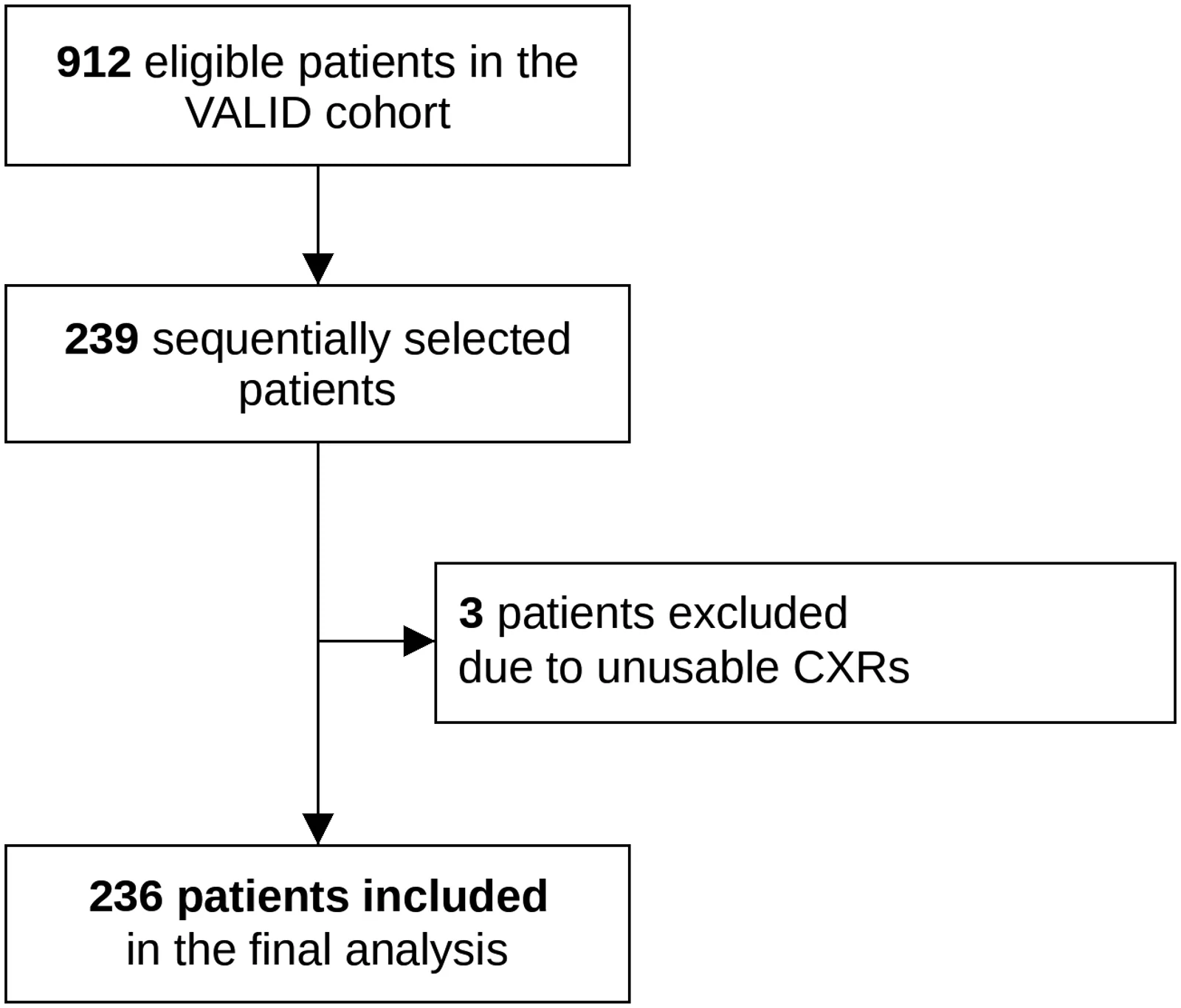
Patient flow. CXR = chest radiographs, VALID = Validating Acute Lung Injury Biomarkers for Diagnosis.

### RALE Scores and Diagnostic Performance

The highest average RALE score was higher in patients with ARDS compared with those without ARDS (29.5 [22.2–34.5] vs. 12.2 [7.5–17.8]; *p* < 0.01; **Fig. 2**). Asymmetry in RALE scores between the two lungs was frequently apparent in individual patients (Figs. 2 and **3**, **center panels**). The RALE score had an excellent diagnostic performance for ARDS with an AUROC of 0.93 (95% CI, 0.90–0.96). The cutoff of 20 yielded optimal discrimination, corresponding to sensitivity and specificity of 85% (76–91%) and 83% (76–89%), and positive and negative predictive values of 76% (67–84%) and 90% (83–94%; Table S2, https://links.lww.com/CCX/B693).

**Figure 2.**
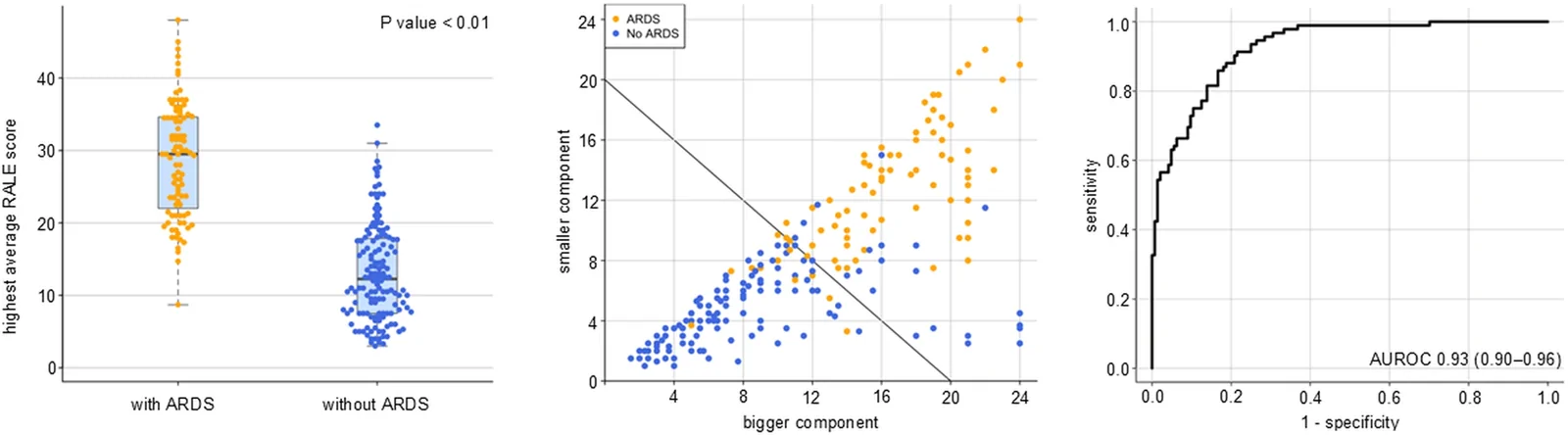
Diagnostic performance of the Radiographic Assessment of Lung Edema (RALE) score for acute respiratory distress syndrome (ARDS). **Left**, Highest average RALE score in patients with ARDS (*orange*) and in patients without ARDS (*blue*). *Boxes* indicate the interquartile range, the *horizontal line* within each *box* the median, and the whiskers the minimum and maximum values; individual patient scores are overlaid as dots. **Center**, Distribution of the RALE score across the two lungs. The *x*-axis shows the score of the higher-scoring lung and the *y*-axis the score of the lower-scoring lung; each *dot* represents one patient (*orange*, ARDS; *blue*, no ARDS). Because the higher score is always plotted on the *x*-axis, all *dots* lie on or below the *line* of equal scores (not shown); *dots* closer to that *line* indicate more symmetric involvement of the two lungs, and *dots* farther below it indicate greater left-right asymmetry. *Dots* above the *solid diagonal line* correspond to a total RALE score of 20 or higher. **Right**, Receiver operating characteristic curve for the discrimination of ARDS by the highest average RALE score obtained from chest radiographs on the first 2 calendar days in the ICU. The area under the receiver operating characteristic curve (AUROC) is shown with its 95% CI.

### Sensitivity Analysis

The highest average RALE score was higher in patients with ARDS compared with those without ARDS, both when using only CXRs of acceptable quality (30 [22–35] vs. 11 [7–17]; *p* < 0.01) and when using CXRs of borderline quality (30 [21–35] vs. 11 [7–17]; *p* < 0.01). The diagnostic performance remained excellent when excluding CXRs of borderline quality (**Fig. 3**).

**Figure 3.**
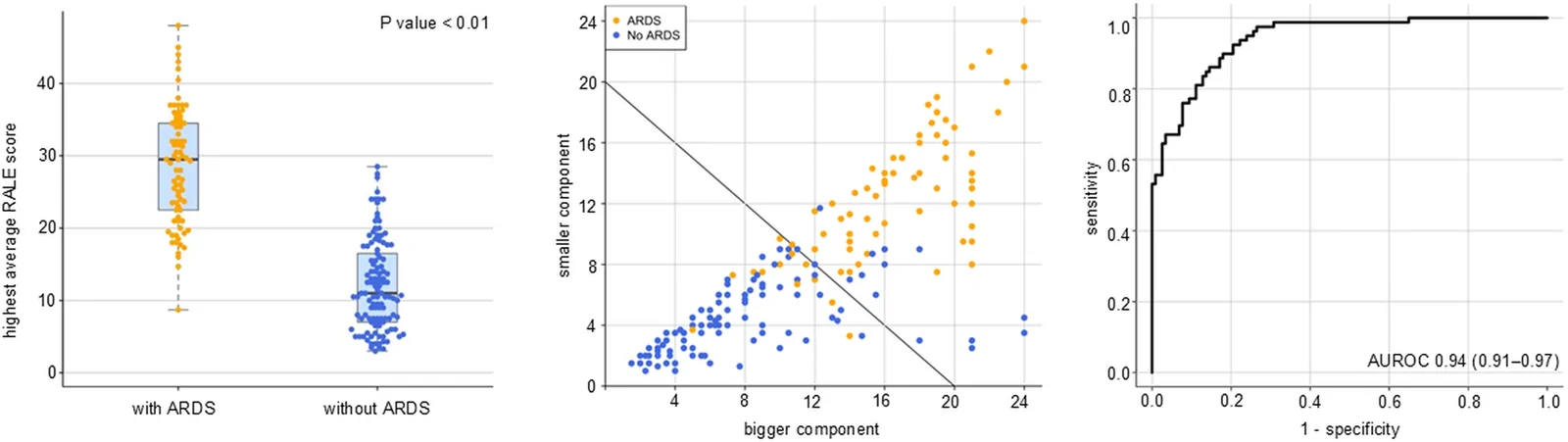
Sensitivity analysis of the diagnostic performance of the Radiographic Assessment of Lung Edema (RALE) score for acute respiratory distress syndrome (ARDS) including only patients with chest radiographs rated as “acceptable” by all scorers. **Left**, Highest average RALE score in patients with ARDS (*orange*) and in patients without ARDS (*blue*). *Boxes* indicate the interquartile range, the *horizontal line* within each box the median, and the whiskers the minimum and maximum values; individual patient scores are overlaid as *dots*. **Center**, Distribution of the RALE score across the two lungs. The *x*-axis shows the score of the higher-scoring lung and the *y*-axis the score of the lower-scoring lung; each *dot* represents one patient (*orange*, ARDS; *blue*, no ARDS). Because the higher score is always plotted on the *x*-axis, all *dots* lie on or below the *line* of equal scores (not shown); *dots* closer to that *line* indicate more symmetric involvement of the two lungs, and *dots* farther below it indicate greater left-right asymmetry. *Dots* above the *solid diagonal line* correspond to a total RALE score of 20 or higher. **Right**, Receiver operating characteristic curve for the discrimination of ARDS by the highest average RALE score. The area under the receiver operating characteristic curve (AUROC) is shown with its 95% CI.

### Interobserver Reliability

Interobserver reliability was excellent and consistent across all rater pairs. All coefficients were computed on the raters’ independent scores, obtained before any reconciliation. The pairwise ICC was 0.87 (95% CI, 0.84–0.89) between raters I and II (*n* = 151), 0.85 (95% CI, 0.80–0.88) between raters II and III (*n* = 69), and 0.81 (95% CI, 0.78–0.84) between raters III and I (*n* = 154; **Fig. 4**). The ICC where all three raters scored the RALE score was 0.85 (95% CI, 0.79–0.90), based on the 69 radiographs scored by all three raters. Discrepancies between readers exceeded the predefined thresholds in 1.3% of radiographs for the total RALE score and in 9.4% of quadrant comparisons.

**Figure 4.**
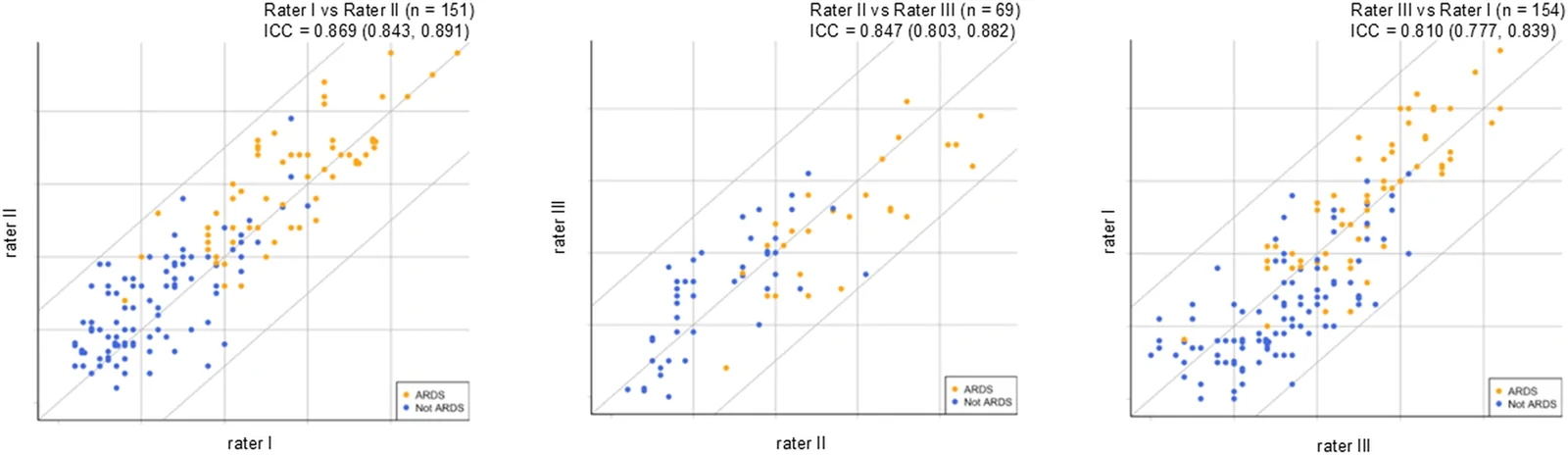
Correlation between the three raters based on their independent scores obtained before reconciliation. Each *dot* represents a pair of scores for a single chest radiograph in patients with acute respiratory distress syndrome (ARDS; *orange dots*) and without ARDS (*blue dots*), with the Radiographic Assessment of Lung Edema score assigned by one rater on the *x*-axis and the score assigned by another rater on the *y*-axis. The *solid diagonal line* through the origin indicates perfect agreement between raters; the *parallel diagonal lines* indicate acceptable differences in scores. ICC = intraclass correlation coefficient.

## DISCUSSION

The findings of this post hoc analysis of VALID can be summarized as follows: 1) the median RALE score was higher in patients with ARDS compared with those without ARDS; and 2) the RALE score had an excellent diagnostic performance for ARDS. Additionally, 3) we recorded substantial asymmetry in RALE scores between the left and right lungs. We observed 4) strong interobserver reliability, and 5) a sensitivity analysis limited to patients with acceptable CXRs confirmed that the RALE score’s diagnostic performance remained robust, suggesting that the results were not biased by radiograph quality.

Strengths include enrollment from the four ICUs of the participating center and ARDS arising from a range of predisposing conditions, most often pneumonia, aspiration, or sepsis, which supports applicability across the causes of ARDS encountered in this population. The reference standard was prespecified and was the endpoint of the original study; unusable CXRs were excluded, all remaining radiographs were scored independently by at least two raters blinded to clinical data and group allocation, and a predefined analysis plan was strictly followed to minimize the risk of bias.

Our findings build upon and extend earlier research demonstrating the potential of the RALE score as a diagnostic tool for ARDS. A single-center study of invasively ventilated ICU patients, both with and without ARDS, reported excellent diagnostic accuracy of the RALE score, although the investigators did not define a universally applicable RALE cutoff (9). Subsequent studies have shown that RALE scores are consistently and significantly higher in patients with ARDS (15, 16). Notably, even in the context of COVID-19, where lung involvement may differ in pattern and severity, the RALE score may be a discriminative marker, with significantly elevated values in patients with COVID-19–associated ARDS compared with those without ARDS (16). Taken together, this growing body of evidence supports the RALE score’s potential utility across a broad spectrum of ARDS presentations, including those due to emerging or atypical causes.

These observations are timely. The most recent global definition of ARDS retained bilateral opacities on chest imaging as a core criterion while acknowledging that both CXR and lung ultrasound are highly interpreter-dependent; formal radiographic scoring systems such as the RALE score were considered but ultimately not incorporated, pending further prospective validation, and were highlighted as a priority for future research (7). By showing that the RALE score identifies ARDS with high accuracy and reproducibility, our findings represent a step toward the validation that such integration would require.

Our findings align with previous research showing strong interrater agreement in RALE score assessment (9, 11, 17). The first study introducing the RALE score reported high concordance between independent observers (8). Subsequent work also demonstrated good reliability, although a third reviewer was sometimes required to resolve discrepancies, indicating that differences can occur in certain cases (15). In our study, substantial discrepancies between readers were similarly resolved through joint reassessment, which produced the values used in the diagnostic analysis. Because such adjudication is not available in routine care, agreement estimates derived after reconciliation would overstate the reproducibility achievable at the bedside; we therefore report the agreement obtained from the raters’ independent readings, which is the estimate relevant to clinical use. That structured feedback and re-reading improve agreement is not specific to our cohort and has been documented in a large multicenter series, in which the ICC rose from 0.85 to 0.93 after a cycle of feedback and independent re-scoring (11); this argues for brief standardized training before the score is applied rather than against the score itself. Even minimal training yields good-to-excellent agreement (18) and expert-led instruction before independent scoring further improves concordance with the trainer (16, 19). This matters in ARDS, where radiographic interpretation is subjective and interobserver variability has long been recognized (2, 3). Our results confirm that the RALE score remained consistent across raters, supporting its use in both research and clinical settings.

We observed marked asymmetry in RALE scores between the two lungs in individual patients, including patients meeting the Berlin definition. This is a descriptive observation, which we did not test; whether regional distribution, between hemithoraces, or between upper and lower quadrants, carries information beyond the summed score warrants a dedicated analysis. This finding is noteworthy and may reflect anatomical, physiologic, or pathologic differences in lung involvement. Although direct comparisons of RALE score by laterality are scarce, a study on unilateral pulmonary edema after mitral valve surgery demonstrated that right hemithorax RALE score and the right-to-left RALE score difference provided high sensitivity and specificity for identifying unilateral edema (20). The RALE score can therefore detect clinically relevant side-specific differences, and quadrant- or hemithorax-specific patterns may carry information beyond the total score.

The optimal RALE score cutoff of 20 identified in our study for the diagnosis of ARDS is higher than that reported in the only previous study that proposed a diagnostic threshold for ARDS based on the RALE score, a cutoff of 10 (9). This discrepancy is likely attributable to methodological differences. In that earlier analysis, the RALE score was derived from a single CXR, the first obtained after initiation of mechanical ventilation. In contrast, our approach considered all CXRs obtained during the first 2 ICU days, selecting the one with the highest average RALE score across raters. This strategy was designed to capture the most severe degree of radiographic edema in the early course of critical illness, which may have resulted in higher overall RALE scores and, consequently, a higher diagnostic threshold for ARDS. Notably, the identification of an optimal RALE score cutoff of 20 strengthens the diagnostic utility of the score by providing a practical, objective threshold for ARDS classification in invasively ventilated patients, facilitating clinical decision-making and potentially supporting earlier intervention.

The appeal of the RALE score lies in its ability to be applied directly to routine CXRs, without requiring additional imaging or reliance on artificial intelligence (AI) algorithms. This simplicity enhances its value in resource-limited settings, where access to advanced imaging or computational tools may be restricted. By leveraging existing diagnostic resources, the RALE score provides a practical, efficient method for assessing pulmonary conditions across diverse healthcare environments. Future work should compare its performance with other modalities, such as lung ultrasound, which can detect bilateral loss of aeration when performed by skilled operators (21) and CT. Multicenter validation is essential to confirm robustness and reproducibility, whereas studies involving multiple readers could clarify interobserver variability and optimize application. Finally, efforts should focus on ensuring generalizability across varied ICU patient populations, broadening the RALE score’s utility in clinical practice.

A key limitation of our study lies in its reliance on a post hoc analysis of the VALID cohort, which itself was defined by rigorous inclusion and exclusion criteria that ensure internal validity but may constrain generalizability (10). Our additional post hoc filters, selecting only invasively ventilated patients with ARDS risk factors and excluding those with trauma or cardiogenic edema, further narrow the population in which the RALE score was evaluated. Our estimates are, in addition, cohort-wide. The study was not powered to compare diagnostic accuracy across predisposing conditions, and stratification by PaO_2_/FiO_2_ is precluded because this variable forms part of the reference standard. Whether the performance of the RALE score differs across clinical phenotypes of ARDS warrants a dedicated analysis in a larger or pooled cohort. We excluded all trauma admissions rather than only those with thoracic injury; a criterion based on thoracic injury severity would have been more discriminating, and we recommend it for future evaluations of the RALE score. A further limitation is structural rather than procedural. Bilateral opacification is one of the four components of the Berlin definition used here as the reference standard, and the RALE score quantifies opacification on the same radiograph. Our estimates of diagnostic accuracy are therefore subject to a degree of incorporation bias and are best read as an upper bound. Three features attenuate this: the radiographic criterion is one of four, the remaining three being independent of the RALE score; the RALE score is a graded measure of the extent and density of opacification rather than the dichotomous judgment of bilaterality; and the raters were blinded to ARDS status. The score is agnostic to laterality, as shown by its ability to identify unilateral pulmonary edema (20), and marked left-right asymmetry was present in our cohort. A high total score is therefore not simply a restatement of the radiographic criterion. Furthermore, our reliance on CXR as the sole imaging modality may have missed abnormalities detectable by lung ultrasound or CT, particularly in patients with borderline or equivocal CXR findings. Future research should address these gaps by applying RALE scoring across broader ICU populations, integrating multimodal imaging, and directly comparing its performance with alternative diagnostic approaches, including AI-based models. Integrating imaging with physiologic and predictive models may improve early risk stratification and support precision medicine in ARDS (22–24).

## CONCLUSIONS

The RALE score accurately identified ARDS in this cohort of invasively ventilated patients and demonstrated excellent interobserver agreement.

## Supplementary Material
