# Supplementary material for: Diagnostic Performance of the Radiographic Assessment of Lung Edema Score for Acute Respiratory Distress Syndrome—Post Hoc Analysis of the “Validating Acute Lung Injury Biomarkers for Diagnosis” Study
Source: Crit Care Explor. 2026 Oct 21;8(10):e1495. doi: 10.1097/CCE.0000000000001495 (PMC13686730; doi:10.1097/CCE.0000000000001495)
Supplement: Supplementary file 1 [file cc9-8-e1495-s001.pdf]

Supplemental Materials

Diagnostic Performance of the RALE Score for ARDS – post hoc analysis of VALID

Supplemental Table S1. The Standards for Reporting of Diagnostic Accuracy Studies (STARD) 2015 checklist.

Section & Topic	No	Item	Reported on page #
**TITLE OR ABSTRACT**			
	**1**	Identification as a study of diagnostic accuracy using at least one measure of accuracy (such as sensitivity, specificity, predictive values, or AUC)	pp. 1,3 — Title; Abstract – pp. 6, 11 — Methods/Results. Study identified as a diagnostic accuracy analysis reporting AUROC, sensitivity, specificity and predictive values.
**ABSTRACT**			
	**2**	Structured summary of study design, methods, results, and conclusions (for specific guidance, see STARD for Abstracts)	pp. 3,4 — Abstract, structured as Objective / Design / Setting / Patients / Interventions / Measurements and Main Results / Conclusions.
**INTRODUCTION**			
	**3**	Scientific and clinical background, including the intended use and clinical role of the index test	p. 5 — Introduction, paragraphs 1–2 (role of bilateral opacities in ARDS diagnosis; RALE as a semi-quantitative radiographic tool and its intended diagnostic use).
	**4**	Study objectives and hypotheses	p. 5 — Introduction, final paragraph (objective: diagnostic accuracy of the RALE score for ARDS; hypothesis of high accuracy; secondary aim of interobserver reliability).
**METHODS**			
Study design	**5**	Whether data collection was planned before the index test and reference standard were performed (prospective study) or after (retrospective study)	p. 6 — Methods – Study design and settings. Post hoc analysis of the prospective observational VALID study; RALE scoring (index test) performed retrospectively on prospectively collected CXRs.
Participants	**6**	Eligibility criteria	pp. 6,7 — Methods – Patients (VALID eligibility criteria plus additional post hoc

			criteria: invasive ventilation ≥2 days and an ARDS risk factor).
	**7**	On what basis potentially eligible participants were identified (such as symptoms, results from previous tests, inclusion in registry)	pp. 7-10 — Methods – Patients and Statistical analysis (ventilated patients with ARDS risk factors, identified from the VALID database).
	**8**	Where and when potentially eligible participants were identified (setting, location and dates)	pp. 6,7 — Methods – Study design and settings (four medical, cardiac, surgical and trauma ICUs at Vanderbilt University Medical Center, Nashville, USA; enrolment from January 2006).
	**9**	Whether participants formed a consecutive, random or convenience series	pp. 7-9 — Methods – Patients / Statistical analysis (consecutive series; eligible patients selected sequentially from the VALID database until the target size was reached).
Test methods	**10a**	Index test, in sufficient detail to allow replication	p. 8 — Methods – RALE score (four-quadrant method; consolidation 0–4 and density 1–3 per quadrant; total 0–48; scoring, quality rating and reconciliation procedure described).
	**10b**	Reference standard, in sufficient detail to allow replication	pp. 5,9 — Introduction/Methods – Endpoint (ARDS per the Berlin definition during the first two days of ventilation).
	**11**	Rationale for choosing the reference standard (if alternatives exist)	pp. 5,9 — Introduction/Methods – Endpoint; Introduction. Berlin definition is the established clinical reference standard for ARDS and was the endpoint of the parent study.
	**12a**	Definition of and rationale for test positivity cut-offs or result categories of the index test, distinguishing pre-specified from exploratory	pp. 9,10 — Methods – Statistical analysis. RALE cut-off of 20 derived from ROC analysis (integer values only); exploratory/data-driven, not pre-specified.
	**12b**	Definition of and rationale for test positivity cut-offs or result categories of the reference standard, distinguishing pre-specified from exploratory	pp. 5,9 — Introduction/Methods – Endpoint (ARDS present/absent per the Berlin definition; pre-specified).
	**13a**	Whether clinical information and reference standard results were available to the performers/readers of the index test	pp. 8,9 — Methods – RALE score (raters scored CXRs while blinded to clinical details and to ARDS status).
	**13b**	Whether clinical information and index test results were available to the assessors of the reference standard	pp. 7-10 — Reference standard (ARDS classification) was established in the parent study independently of and prior to the post

			hoc RALE scoring; index test results were therefore unavailable to those assessors.
Analysis	**14**	Methods for estimating or comparing measures of diagnostic accuracy	pp. 9,10 — Methods – Statistical analysis (ROC curves and AUROC; sensitivity, specificity, PPV and NPV at the optimal cut-off; AUROC interpretation bands).
	**15**	How indeterminate index test or reference standard results were handled	pp. 8-10 — Methods – RALE score / Statistical analysis (CXRs rated 'unusable' excluded; 'borderline' quality examined in a sensitivity analysis).
	**16**	How missing data on the index test and reference standard were handled	pp. 8-11 — Methods / Results (three patients with unusable CXRs were excluded; Figure 1).
	**17**	Any analyses of variability in diagnostic accuracy, distinguishing pre-specified from exploratory	pp. 9,10 — Methods – Statistical analysis (sensitivity analysis by CXR quality; interobserver reliability by ICC, pairwise and three-rater).
	**18**	Intended sample size and how it was determined	pp. 9,10 — Methods – Statistical analysis (power calculation: assumed 40% ARDS prevalence, 80% power, α 0.05; 228 required, 239 selected to allow for unusable CXRs).
**RESULTS**			
Participants	**19**	Flow of participants, using a diagram	pp. 11,12 — Results – Patients; Figure 1 (patient flow).
	**20**	Baseline demographic and clinical characteristics of participants	pp. 6,7,11,12 — Methods/Results – Patients; Table 1.
	**21a**	Distribution of severity of disease in those with the target condition	pp. 6,7,11,12 — Methods/Results; Table 1 (APACHE II, SAPS II, distribution of PaO_2/FiO_2 among patients meeting the Berlin definition).
	**21b**	Distribution of alternative diagnoses in those without the target condition	pp. 11,12 — Results – Patients; Table 1 (ARDS risk factors/causes).
	**22**	Time interval and any clinical interventions between index test and reference standard	pp. 6–9 — Methods – RALE score / Endpoint. Index test and reference standard both ascertained within the first two ICU days; no intervening interventions relevant.
Test results	**23**	Cross tabulation of the index test results (or their distribution) by the results of the reference standard	pp. 11,12 — Results – RALE scores and diagnostic performance; Figure 2,3 (score

			distributions); Supplemental Table S2 (2×2 cross-tabulation at the cut-off of 20).
	**24**	Estimates of diagnostic accuracy and their precision (such as 95% confidence intervals)	pp. 11,12 — Results – RALE scores and diagnostic performance (AUROC 0.93 [0.90–0.96]; sensitivity 85% [76–91], specificity 83% [76–89], PPV 76% [67–84], NPV 90% [83–94]).
	**25**	Any adverse events from performing the index test or the reference standard	Not applicable. The RALE score is applied to routine CXRs; no adverse events from the index test or reference standard.
**DISCUSSION**			
	**26**	Study limitations, including sources of potential bias, statistical uncertainty, and generalisability	pp. 16,17 — Discussion, limitations paragraph (post hoc analysis of a single-center VALID cohort; incorporation bias, in that bilateral opacities contribute to both the index test and the reference standard; restricted study population (invasive ventilation and a risk factor for ARDS); exclusion of all trauma admissions; cohort-wide estimates not stratified by subgroup; chest radiography as the sole imaging modality).
	**27**	Implications for practice, including the intended use and clinical role of the index test	pp. 15–18 — Discussion and Conclusion (objective threshold for ARDS classification; value in resource-limited settings; need for prospective, multimodal validation).
**OTHER INFORMATION**			
	**28**	Registration number and name of registry	pp. 3,4,6 — Registration/Methods – Vanderbilt IRB protocol number 051065; not registered in a clinical-trial registry.
	**29**	Where the full study protocol can be accessed	pp. 3,4,6 — Registration/Methods – Study design and settings (VALID methods previously published; ref 10).
	**30**	Sources of funding and other support; role of funders	pp. 2, 4 — Funding – Disclosures (no additional funding for this post hoc analysis; none declared).

Supplemental Table S2. Cross-tabulation of the RALE score (cutoff of 20) against the diagnosis of ARDS according to the Berlin definition (reference standard).

RALE score	ARDS	No ARDS	Total
≥ 20	78	24	**102**
< 20	14	120	**134**
**Total**	92	144	**236**

Values are numbers of patients. ARDS, acute respiratory distress syndrome; RALE, Radiographic Assessment of Lung Edema. The diagnostic accuracy estimates derived from this table are reported in the main text.

Selection of the optimal RALE score cutoff

At a cutoff of 20, the RALE score yielded optimal discrimination for ARDS, with a sensitivity of 85% (95% CI 76–91) and a specificity of 83% (95% CI 76–89), and positive and negative predictive values of 76% (95% CI 67–84) and 90% (95% CI 83–94), respectively.

The optimal cutoff was determined as follows:

- Only integer cutoff values were considered, because the RALE score is itself integer-valued;
- Sensitivity and specificity were weighted equally;
- Two complementary criteria were applied: the maximum of the average of sensitivity and specificity (equivalent to the Youden index), and the minimum distance to perfect classification in ROC space, defined as $(1 - \text{sensitivity})^2 + (1 - \text{specificity})^2$. Both criteria identified a cutoff of 20.
